# Editorial: Mutational breeding: from induced mutations to site-directed mutagenesis

**DOI:** 10.3389/fpls.2024.1511363

**Published:** 2024-11-11

**Authors:** Søren K. Rasmussen, Shri Mohan Jain

**Affiliations:** ^1^ Retired, Roskilde, Denmark; ^2^ Department of Agricultural Sciences, University of Helsinki, Helsinki, Finland

**Keywords:** CRiSPR/Cas, irradiation, breeding nursery, genetic resource, grain crop, climate change, mutant variety, fruit tree

## Introduction

Plant breeding utilizes variation from gene mutations to develop new cultivars in a wide range of crop plants, ornamentals, and fruit trees, as monitored by IAEA (2024), and for more basic genomic research in Arabidopsis. A high number of traits have been modified by induced plant mutations, such as resistance to pathogens (Suprasanna and Jain, 2023), flower colors of ornamentals (Suprasanna and Jain, 2022), lignin content (Christensen and Rasmussen, 2019), and chemical-nutritional composition of mature seeds. The desired traits are obtained from spontaneous/natural occurring mutations or, more often, by chemically or physically induced mutations or transposable elements as the biological agents. All these agents are believed to randomly hit the plant genome in an unbiased way without the need for pre-knowledge of DNA sequences in order to be successful in breeding and basic genome research. A breakthrough came with the Nobel prize-winning CRISPR/Cas method in chemistry 2020 that, in contrast to the above, allows site-directed mutagenesis to be carried out by taking advantage of known DNA sequences. Various oligo-nucleotide-directed mutations in chromosomal DNA have been used as well. In light of climate change, the future challenge will be to combine the progress made and multitude of molecular genetic tools available with mutational breeding to improve the multi-gene quantitative traits for tolerance to abiotic stress.

The collection of research articles in this Research Topic on mutational breeding discusses the chemically induced mutation used in three important grain crops, namely wheat, barley, and bean, as well as physically induced mutations by irradiation in pear fruit tree, in a perennial grass species, and in the model plant Arabidopsis. Self-compatibility is a desired trait in the breeding of fruit trees such a triploid pear (*Pyrus pyrofolia*); Nishio et al. develop a strategy termed “selective pollination” using mutant pollen obtained by gamma-irradiation to produce a duplication of a short chromosomal segment in the S-locus. This locus is found in many plant species and thus the results have implications for breeding in general. Irradiation-induced deletions of chromosomal segments in Arabidopsis were used for functional analysis of tandem-arrayed genes by (Ishii et al.). Repeated genes and gene families are a characteristic of plant genomes, and they create redundancy in gene functions. The paper gives a good introduction to irradiation methods and how such deletion of segments carrying entire genes by mutations may serve as a tool in crop plant breeding. Sanjaya et al. use heavy-ion beam irradiation of Arabidopsis to show that basic genome research may have application in biotechnology across species. They report on a mutation in the intron/exon border sequences that can activate a cryptic splice site. The authors then discuss the effectiveness of heavy-ion beam irradiation to expand the scope of obtaining unexpected and valuable mutants. For most crop plants, the genomic sequence is available and mutations can be searched for in regulatory elements as well as the genes they act on. In this way, a series of mutations in the regulatory sequences providing different expression levels can be identified and used in breeding programs where a fine tuning of specific enzymes, *in casu* phytase, is in demand for improving grain nutritional quality or nutrient uptake from the soil. Madsen et al. use the in-house FIND-IT platform (Knudsen et al., 2022) to screen a chemical-induced mutation population in barley (*Hordeum vulgare* L.) for alleles in the promotor sequence of a phytase gene and show that these alleles provide different strengths of expression of the phytase gene. Tomlekova et al. use chemical mutagenesis of common bean (*Phaseolus vulgaris* L.) to develop a nursery for a regional breeding program with the aim of adapting the bean to the agroclimatic zone of the region. They observe large duplications of chromosome fragments which led to an increase in genome size. How this genome expansion may lead to increased productivity of the bean mutant lines is discussed. Makebe et al. describe a number of distinct mutant lines of hexaploid wheat (*Triticum aestivum* L.) that have been propagated to the M5 generation and through this provide novel wheat genotypes that combine yield-influencing agronomic traits, enhanced biomass allocation and root systems, and high grain yield potential to improve wheat production under drought-stressed and water-limited environments in South Africa and regions with similar agro-climatic conditions. All these traits are central targets for adapting cultivars to climate change. Zhang et al. use ion beam irradiation to create a mutant germplasm in the perennial centipede grass (*Eremochloa ophiuroides*). Centipede grass is vegetatively/asexually propagated and only after a few years can flowering be induced and a small number of seeds collected. Therefore, microsatellite markers were used on the putative M1 mutant lines that have been vegetatively propagated in order to demonstrate significant changes in DNA profile as a marker for mutation efficiency.

## Conclusion

When combined with the newest methods in DNA sequencing and marker-assisted selection, molecular and mutational breeding offer new possibilities for creating biodiversity that can be utilized in plant improvements. Access to the demanding radiation facilities (Ma et al., 2021) proved its importance for creating mutations in crop plants as well as for basic genome research. Mutagenizing by using chemicals, on the other hand, is much less demanding in terms of access to facilities and skills and is available worldwide. Together with site-directed mutagenesis, the well-established induced-mutation methods will be key to crop improvement for resilience to high temperatures and drought as a consequence of climate change and for the benefit of the society.

## References

[B1] Christensen C. S. L. Rasmussen S. K. (2019). Low lignin mutants and reduction of lignin content in grasses for increased utilisation of lignocellulose. Agronomy 9, 256. doi: 10.3390/agronomy9050256

[B2] IAEA. (2024). Mutant variety database. Available online at: https://nucleus.iaea.org/sites/mvd/SitePages/Home.aspx (Accessed October 11, 2024).

[B3] Knudsen S. Wendt T. Dockter C. Thomsen H. C. Rasmussen M. Egevang Jørgensen M.. (2022). FIND-IT: Accelerated trait development for a green evolution. Sci. Adv. 8, eabq2266–eabq2266. doi: 10.1126/sciadv.abq2266 36001660 PMC9401622

[B4] Ma L. Kong F. Sun K. Wang T. Guo T. (2021). From classical radiation to modern radiation: past, present, and future of radiation mutation breeding. Front. Public Health 9. doi: 10.3389/fpubh.2021.768071 PMC8725632 34993169

[B5] Suprasanna P. Jain S. M. (2022). Biotechnology and induced mutations in ornamental plant improvement. Acta Hortic. 1334, 1–12. doi: 10.17660/ActaHortic.2022.1334.1

[B6] Suprasanna P. Jain S.M. (Eds.) (2023). Mutation breeding for sustainable food production and climate resilience (Singapore: Springer). Available at: https://link.springer.com/book/10.1007/978-981-16-9720-3.

